# Anti-*Pterobothrium heteracanthum* (Trypanorhyncha: Pterobothriidae) IgG in human serum samples

**DOI:** 10.1590/S1984-29612024071

**Published:** 2024-11-22

**Authors:** Maurício Afonso Verícimo, Israel Figueiredo, Sérgio Carmona de São Clemente, Michelle Cristie Gonçalves da Fonseca, Marcelo Knoff, Danuza Pinheiro Bastos Garcia de Mattos

**Affiliations:** 1 Laboratório de Imunobiologia das Doenças Infecciosas e Granulomatosas, Instituto de Biologia, Universidade Federal Fluminense – UFF, Niterói, RJ, Brasil; 2 Hospital Universitário Antônio Pedro, Departamento Materno-Infantil, Universidade Federal Fluminense – UFF, Niterói, RJ, Brasil; 3 Laboratório de Inspeção e Tecnologia de Pescado, Departamento de Tecnologia de Alimentos, Faculdade de Veterinária, Universidade Federal Fluminense – UFF, Niterói, RJ, Brasil; 4 Laboratório de Helmintos Parasitos de Vertebrados, Instituto Oswaldo Cruz, Fundação Oswaldo Cruz – Fiocruz, Rio de Janeiro, RJ, Brasil; 5 Departamento de Microbiologia e Parasitologia, Instituto Biomédico, Universidade Federal Fluminense – UFF, Niterói, RJ, Brasil

**Keywords:** Cestode, plerocerci, fish parasite, ELISA, human serum, Cestoide, plerocercos, parasito de peixe, ELISA, soro humano

## Abstract

Some fish parasites can cause a variety of symptoms in humans, including allergies. This was a cross-sectional study based on interviews, serum analysis by ELISA for anti–*Pterobothrium heteracanthum* IgG and a statistical evaluation. Four individuals were seroreactive (6.25%), with no association with fish handling (p = 1.000) or with ingestion more than twice a week (p = 0.232). There was a significant association (p = 0.032) between reactivity and the absence of allergy symptoms. Seroreactivity against *P. heteracanthum* in humans was detected, but was not associated with the amount of fish ingested, handled, or with allergic complaints.

## Resumo

Alguns parasitos de peixes podem causar uma variedade de sintomas em humanos, incluindo alergias. Este foi um estudo transversal baseado em entrevistas, análise sérica por ELISA para IgG anti–*Pterobothrium heteracanthum* e avaliação estatística. Quatro indivíduos foram sororreativos (6,25%), sem associação com o manuseio dos peixes (p = 1,000) ou com a ingestão maior que duas vezes por semana (p = 0,232). Houve associação significativa (p=0,032) entre reatividade e ausência de sintomas alérgicos. A sororreatividade contra *P*. *heteracanthum* em humanos foi detectada, mas não foi associada à quantidade de peixe ingerido, manuseado ou a queixas alérgicas.

Fishing is one of the oldest forms of feeding the human population and continues to be of great importance. World production by capture and the aquaculture process provided 214 million tons in 2020, of which 157 million tons were for human consumption. The world average of apparent per capita consumption was 20.2 kg for the same year (FAO, 2022).

Despite the nutritional benefits associated with fish consumption, allergic manifestations can be developed mainly for three reasons: the presence of histamine in the food; immunological reactions against parvalbumin; or parasite antigens present in the flesh (Prester, 2016). Not all parasites that inhabit fish are capable of infecting the human organism, but they still have the potential to harm human health, since they could cause allergies in individuals who have been sensitized at some point in their lives (Nieuwenhuizen et al., 2006; Pelayo et al., 2009; Mattos et al., 2015).

*Pterobothrium heteracanthum* Diesing, 1850 (Trypanorhyncha: Pterobothriidae) are cestodes commonly found parasitizing fish in Brazil (Eiras et al., 2016), but reports of parasitism by trypanorhynch cestodes in humans are rare and of short duration (Kikuchi et al., 1981; Fripp & Mason, 1983). However, several studies have shown immune sensitization by the antigens of these cestodes in humans and in experimental animal models (Rodero & Cuéllar, 1999; Vázquez-López et al., 2001, Gòmez-Morales et al., 2008; Pelayo et al., 2009; Mattos et al., 2015, 2024). The aim of this research was to verify the presence of anti–*Pterobothrium heteracanthum* plerocerci IgG antibodies in serum samples from healthy human volunteers, and to establish any association between serum reactivity with a high fish intake, handling or a history of allergic symptomatology.

In this cross-sectional study, a questionnaire was applied, and blood samples were collected and analyzed. The study was explained and informed consent obtained before any subject participated in the study.

The samples were obtained from individuals in a military unit of the municipality of Niterói, State of Rio de Janeiro, Brazil. All subjects underwent regular medical examinations and were subject to various disease prevention protocols, including the prevention of intestinal parasites, as required by the public security auxiliary force. Unhealthy individuals, those who did not agree to sign the term of free and informed consent, those who did not have all the data, those who were taking immunosuppressants and those who did not eat fish, were excluded, leaving 64 participants.

Data about the frequencies of handling and consuming fresh, frozen, raw, or lightly cooked fish; and the history of allergic events (skin, respiratory, or gastrointestinal manifestations) were obtained from a structured questionnaire.

Blood samples (5 mL) were obtained from the cubital vein and the sera collected from the volunteers were stored at -20 °C until analyzed.

Plerocerci from *P. heteracanthum* were obtained using scissors and tweezers during fish evisceration and filleting at the Laboratório de Inspeção e Tecnologia de Pescado, Faculdade de Veterinária -UFF (Fish Inspection and Technology Laboratory, Faculty of Veterinary). The fish species used as the source of the parasites was *Micropogonias furnieri* (Desmarest, 1823) (Withemouth croaker), widely traded by the State of Rio de Janeiro fishmongers. Once identified, the parasites were disintegrated in a Potter homogenizer to obtain the total somatic antigens. The complete procedure for obtaining the crude parasite extract was made (Mattos et al., 2015).

The presence of immunoglobulin G (IgG) was measured by ELISA. The optical densities (ODs) of the IgG were analyzed (Figueiredo et al., 2013). Briefly, 50 μL of *P*. *heteracanthum* crude extract solution containing 28 μL of protein/mL was added to each well of a 96-well microplate (MaxiSorp^™^ Nunc^®^, Thermo Fisher Scientific^®^, USA) and incubated overnight at 4 °C. After washing with PBS–Tween (PBS-T), the plates were blocked with 1% PBS-gelatin (PBS–G) for 2 hours at room temperature. Then 50 μL of each serum (duplicates), diluted 1:200 v/v in PBS–G, was added to each well. After 2 hours incubation at 37 °C, the plates were washed with PBS–T and 50 μL of mouse HRP anti–human IgG (Invitrogen^™^, Thermo Fisher Scientific^®^, USA) added to each well according to the manufacturer's instructions, followed by another hour of incubation at 37 °C. Finally, after one last wash, the reaction was revealed by the addition of diluted O–phenylenediamine and H_2_O_2_ in citrate phosphate buffer (pH 5.0). The reaction was stopped using 2N sulfuric acid after 20 minutes. The individual optical density of each well was read using an Anthos 2010 (Biochrom^®^, UK) automatic microplate reader at 490 nm and the results expressed as the average of each duplicate. The cut-off level for positive reactivity was established by calculating three times the 20–well average optical densities of the ELISA reaction described above, replacing human serum with PBS–T. The average for the cut off calculation was defined using the optical density values ​​of the wells that did not receive test serum, obtaining an approximate value of 0.023. The mean value was multiplied by four to obtain the cut off value of approximately 0.092. All optical density values ​greater than or equal to the cut off value were considered indicative of reactivity. This continuous variable, IgG optical density, was analyzed by the ROC curve to define the best sensitivity and specificity.

Binary categorical variables were used to mount the model, such as serum reactivity to *P. heteracanthum* antigens (0 for nonreactive, 1 for reactive), frequencies of handling (0 for not present, 1 for present), frequencies of consuming fresh, frozen, raw or lightly cooked fish (0 for not present, 1 for present) and allergic complaints (0 for not present, 1 for present).

Contingency tables were developed to assess the association between the presence of reactive IgG anti–*P. heteracanthum* responses (0/1) and high fish intake (≥ twice/week) (0/1), ingestion of raw fish (0/1), and the presence of allergic manifestations (Fernández de Corres et al., 2001). The relationship between the categorical variables was tested using a chi-squared analysis and adjusted by Fisher’s exact test. A Generalized Linear Model was used to analyze relationships between the categorical variables. Sensitization to *P. heteracanthum* was the dependent variable, while frequency of fish intake, frequency of handling and the interaction between handling and intake were the independent variables. For all hypotheses, the significance level tested was set at 95%. The data were processed using the SPSS 22.0 statistical package (SPSS, IBM^®^, USA).

Of the 64 individuals analyzed, only 30 (46.9%) reported handling fish. Most claimed to have an ingestion frequency of once or less times a week (49/64 – 76.6%) and did not eat raw fish (52/64 – 81.3%). Only 15 individuals reported eating fish twice or more times a week and only 12 reported eating raw fish. A history of allergies was present in most interviews (36/64 –56.3%).

Only four individuals were labeled as reactive to the *P. heteracanthum* crude extract antigens (4/64 – 6.25%). The mean optical density of anti–*P. heteracanthum* IgG negative sera was 0.045 (95% CI: 0.041 / 0.050; SD: 0.018), whereas for reactors it was 0.123 (95% CI: 0.113 / 0.133; SD: 0.006). When correlating the results and interviewing the reactor individuals, two reported handling fish and two did not (p = 1.000). The same distribution was verified in the frequency of intake, where two individuals ate fish more than twice a week and two did not (p = 0.232). In contrast, it was clear that the four reactors to the *P. heteracanthum* crude extract did not ingest raw fish (p = 1.000) and that there was a significant association (p = 0.032) between reactivity and no allergic complaints, where the four claimed to have no history of allergic phenomena.

A multivariate analysis confirmed there was no relationship between fish handling, frequency of fish intake and serum IgG reactivity (Table 1).

**Table 1:** Generalized Linear Model used to analyze relationships between serum reactivity to *Pterobothrium heteracanthum* antigens and frequency of handling/fish intake frequencies.

Parameter	B	Standard error	95% confidence limits (Wald)		Hypothesis test			Exp (B)	95% confidence limits (Wald) for Exp (B)	
			Lower	Upper	Chi-square (Wald)	gl	Sig.		Lower	Upper
(Intercept)	3.258	1.0190	1.261	5.255	10.222	1	0.001	26.000	3.528	191.597
Fish handling=1	-0.214	1.4443	-3.044	2.617	0.022	1	0.882	0.808	0.048	13.698
Fish handling=0	0^a^							1		
Fish intake=1	-1.466	1.4850	-4.377	1.444	0.975	1	0.323	0.231	0.013	4.238
Fish intake=0	0a							1		
Fish handling=1* Fish intake=1	0.368	2.0966	-3.741	4.477	0.031	1	0.861	1.444	0.024	87.964
(Scale)	1b									

Dependent variable: Reactivity (1); Model: (Intercept), fish handling, fish intake, fish handling * fish intake,

a. Set to zero because this parameter is redundant. (Interactions: Fish handling=1* Fish intake=0; Fish handling=0* Fish intake=0; Fish handling=0* Fish intake=1)

b. Set at listed value.

The identification of serum reactivity in healthy people was well characterized, considering that hypersensitivity in humans also involves the formation of IgA, IgM and IgE, the latter being the most characteristic for allergic conditions. Despite the lack of verifying all the immunoglobulins, this study added the information that the presence of allergic complaints did not prevail among individuals who demonstrated reactivity to the crude *P. heteracanthum* extract.

There are no reports of direct aggression or allergic manifestations by trypanorhynchs in humans as seen by the quantity and variety of nematode infections (Zanelli et al., 2017). Perhaps the type of parasitic behavior outside the traditional host is not as risky, although it is well known that while alive, trypanorhynch metacestodes are not digested by the digestive juices (Magalhães et al., 2012).

In studies using the murine model it was observed that the crude extract of *P. heteracanthum*, when inoculated into BALB/c mice, was able to sensitize the animal, and the presence of circulating immunoglobulin E (IgE) and IgG antibodies was detected by ELISA. The specificity of these immunoglobulins for the parasite in question was tested by the Immunoblot technique, thus proving that the antigens administered were able to generate reactivity through the production of IgE and IgG with a specific recognition region (Mattos et al., 2015), also indicating the allergenic capacity that molecules of trypanorhynchs possess in experiments with murine models (Rodero & Cuéllar, 1999; Vázquez-López et al., 2001; Gòmez-Morales et al., 2008; Mattos et al., 2024).

The presence of trypanorhynch metacestodes in edible fish is a well-known reality along the coast of the State of Rio de Janeiro (Eiras et al., 2016), and consequently it is not surprising to find 6.15% seroreactivity in humans. Analysis on the Spanish population of Madrid, tested specific antibody levels by ELISA and found that reactivity was 15.7% (IgG) in the 305 sera analyzed (Pelayo et al., 2009). This research also showed that seropositivity was not prevalent among fresh fish consumers and was not higher in those with a high frequency of fish consumption.

Some issues must be raised. The cut-off in optical density values ​​turned out to be stricter than those defined by a previous study (Pelayo et al., 2009), who claimed to use a low cut-off to obtain more positive results, sufficient to assess the risk factors from statistical treatments. The cut-off established in the present study was higher than that of the Spanish study (Pelayo et al., 2009) and when analyzed by the ROC curve, it was observed that the stipulated value corresponded to 100% positive identification (sensitivity = 1.000) and a 1.7% chance of false positivity (specificity = 0.017). It is also important to verify the specificity of immunoglobulin (Ig) to the antigen studied using another method, such as Immunoblot, since although very sensitive, the ELISA technique could present cross-reactivity and generate false positive results.

In conclusion, it was possible to identify serum reactivity in healthy humans with the detection of anti-*P. heteracanthum* IgG antibodies, but this reactivity was not associated with the amount of fish ingested or handled, nor with the presence of allergic complaints.

This was the first report of seroreactivity to *P*. *heteracanthum* in humans, providing introductory data. New studies are desirable to expand the sample number and analyzes in order to better understand the potential risk of this parasite in humans.
